# The *Magnolia* Bioactive Constituent 4-O-Methylhonokiol Protects against High-Fat Diet-Induced Obesity and Systemic Insulin Resistance in Mice

**DOI:** 10.1155/2014/965954

**Published:** 2014-05-29

**Authors:** Zhiguo Zhang, Jing Chen, Xin Jiang, Jian Wang, Xiaoqing Yan, Yang Zheng, Daniel J. Conklin, Ki-Soo Kim, Ki Ho Kim, Yi Tan, Young Heui Kim, Lu Cai

**Affiliations:** ^1^Department of Cardiology & Radiation Oncology, First Hospital of Jilin University, Changchun 130021, China; ^2^The Chinese-American Research Institute for Diabetic Complications, Wenzhou Medical University, Wenzhou 325035, China; ^3^Department of Pediatrics, Kosair Children Hospital Research Institute, University of Louisville, Louisville, KY 40202, USA; ^4^College of Bioengineering, Chongqing University, Chongqing 400044, China; ^5^Diabetes and Obesity Center, University of Louisville, Louisville, KY 40202, USA; ^6^Bioland Biotec Co., Ltd., Zhangjiang Modern Medical Device Park, Pudong, Shanghai 201201, China; ^7^Bioland R&D Center, 59 Songjeong 2-gil, Byeongcheon, Dongnam, Cheonan, Chungnam 330-863, Republic of Korea

## Abstract

Obesity is caused by a combination of both genetic and environmental risks. Disruption in energy balance is one of these risk factors. In the present study, the preventive effect on high-fat diet- (HFD-) induced obesity and insulin resistance in mice by *Magnolia* bioactive constituent 4-O-methylhonokiol (MH) was compared with *Magnolia officinalis* extract BL153. C57BL/6J mice were fed by normal diet or by HFD with gavage-administered vehicle, BL153, low-dose MH, and high-dose MH simultaneously for 24 weeks, respectively. Either MH or BL153 slightly inhibited body-weight gain of mice by HFD feeding although the food intake had no obvious difference. Body fat mass and the epididymal white adipose tissue weight were also mildly decreased by MH or BL153. Moreover, MH significantly lowered HFD-induced plasma triglyceride, cholesterol levels and activity of alanine transaminase (ALT), liver weight and hepatic triglyceride level, and ameliorated hepatic steatosis. BL153 only significantly reduced ALT and liver triglyceride level. Concurrently, low-dose MH improved HFD-induced hyperinsulinemia and insulin resistance. Furthermore, the infiltration of mast cells in adipose tissue was decreased in MH or in BL153 treatment. These results suggested that *Magnolia* bioactive constituent MH might exhibit potential benefits for HFD-induced obesity by improvement of lipid metabolism and insulin resistance.

## 1. Introduction

Obesity is a medical condition due to a combination of genetic and environmental risk factors and is reaching epidemic prevalence in developed and developing countries. In 2008, approximately 1.46 billion people were overweight and 502 million people were obese in the world [1]. It is predicted that 51% of the United States population will be obese (BMI > 30) and 11% severely obese by 2030 [2]. Obesity is becoming a well-known risk factor for a number of chronic diseases, including diabetes, cardiovascular diseases, and cancer, and people die from complications of overnutrition and disruption in energy balance every year [3]. It is thought that, in most cases, obesity results from a combination of excessive caloric intake, availability of energy-dense meals, and sedentary behavior. The development of animal models is necessary for investigating the underlying molecular mechanisms of obesity and its pathophysiological effects and for developing new treatments of obesity [4]. The mouse model of high-fat-diet (HFD) feeding-induced obesity has become one of the most important tools for researching the interplay of HFD and the development of obesity. Meanwhile, these models are also used to search for effective therapeutics for obesity [5–7]. As common species of mice used in research, C57BL/6J mice were susceptible of being diet-induced obese compared with other species of mice such as FVB [8] and we have already established a HFD-induced obesity model in C57BL/6J mice [9]. Therefore, C57BL/6J mice were chosen in the present study.

**Figure 1:**
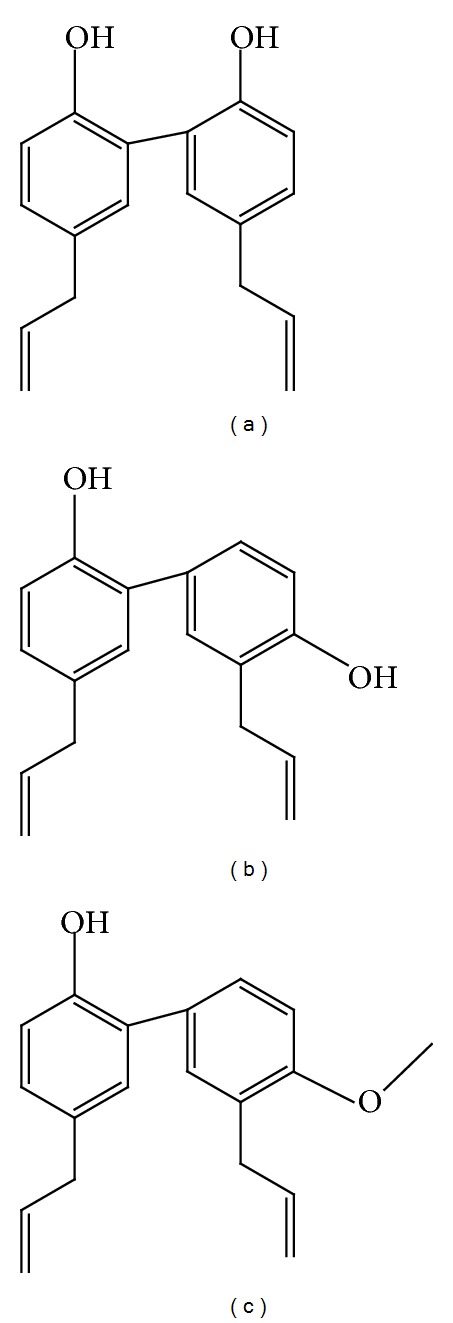
The structures of main components of* Magnolia* extract. (a) Magnolol (MG), (b) honokiol (HK), and (c) 4-O-methylhonokiol (MH).

As a traditional medicine,* Magnolia* has been used to treat gastrointestinal disorders, anxiety, and allergic disease in Asian countries for several centuries.* Magnolia* bark was reported to contain several bioactive compounds, mainly including magnolol (MG), honokiol (HK), 4-O-methylhonokiol (MH), and obovatol, which have diverse functions (Figure 1) [10]. A 6-week pilot clinical study in premenopausal female adults showed that the* Magnolia officinalis* extract reduced evening cortisol levels, systolic blood pressure, and possibly perceived stress, thereby helping to stabilize body-weight [11]. It is found that extracts from* Magnolia kobus* and* Magnolia ovata* or their active components MG and HK also have anti-inflammatory effects in murine macrophage-like cell, human monocytic cell, and mice, respectively [12–14]. In our previous study, we observed that* Magnolia officinalis* extract BL153 at both doses of 5 mg/kg and 10 mg/kg partially attenuated obesity-associated renal and cardiac lipid accumulation, inflammation, oxidative stress, apoptosis, and structural and functional changes; and also partially prevented liver damage in HFD-induced obese mice [9, 15, 16]. Due to the quantitative and qualitative differences of ingredients and possible residual of magnocurarine-like compounds from* Magnolia* bark of different* Magnolia* species, extract process, or growing areas [10, 17], there could exist a diversity of effects of* Magnolia *extracts on treatment. Thus, we investigated whether pure bioactive components were more effective in preventing HFD-induced obesity than the primary* Magnolia officinalis* extract.

Several studies have focused on the pharmacological features of MG or HK such as anti-inflammatory [13], antioxidative stress [18], and cardiovascular protective attributes [19]. It is reported that MG reduced fasting blood glucose and plasma insulin levels in type 2 diabetic rats [20] and increased the glucose uptake in 3T3-L1 adipocytes [21, 22]. In addition, both HK and MG stimulated glucose uptake in insulin-sensitive and insulin-resistant murine and human adipocytes via an insulin signaling pathway [21] and protected tissues and cells against a variety of oxidative stressors [23]. It was also reported that MH, another major bioactive component of* Magnolia* extracts, had anti-inflammatory properties via inhibition of NF-*κ*B pathway in macrophage raw 264.7 cells [24] and inhibited memory impairment and neuronal toxicity induced by beta-amyloid and colon tumor growth in mice [25, 26]. However, it is not clear whether MH can improve HFD-induced obesity and insulin resistance. In the present study, C57BL/6J mice were used to investigate whether MH ameliorates HFD-induced obesity and insulin resistance via anti-inflammation, reducing lipid accumulation, or improving hepatic steatosis. In order to analyze the efficacy of MH on improving lipid profile and insulin resistance induced by HFD, BL153 was used as a comparable reference at the dose of 5 mg/kg which was observed to efficiently improve HFD-induced damages in kidney, heart, and liver [12–14] and almost equal to MH at the dose of 0.5 mg/kg according to the content of MH in BL153 as mentioned in following part.

## 2. Materials and Methods

### 2.1. *Magnolia* Extract (BL-153) and BL-153 Bioactive Constituent 4-O-Methylhonokiol (MH)

BL153 and MH were provided by Bioland Co., Ltd., Chungnam, Korea. A voucher specimen was deposited at the Herbarium of Chungbuk National University, Chungbuk, Korea (voucher specimen # CNBU2009006). The air-dried bark of* Magnolia officinalis* (3 kg) was extracted twice with 95% (v/v) ethanol for 3 days at room temperature. After filtration through the 400-mesh filter cloth, the filtrate was filtered again through filter paper (Whatman, no. 5) and concentrated under reduced pressure to obtain viscous dark-brown residue (360 g, BL153). The combined extract was suspended in H_2_O and the aqueous suspension was extracted with *n*-hexane, ethyl acetate, and *n*-BuOH, respectively. The *n*-hexane layer was evaporated to dryness to give a residue, which was chromatographed on silica gel with *n*-hexane: ethyl acetate (9 : 1) gradient to yield a crude fraction that included MH. The ethanol extract of BL153 was analyzed by HPLC to ensure mainly that it was containing 10.2% of 4-O-methylhonokiol (lot no. MBLEH-052140). This fraction was repeatedly purified by silica gel chromatography using *n*-hexane: ethyl acetate as the eluent to obtain pure MH (purity > 95.45%). BL153 and MH were prepared for gavage solution as 0.5% ethanol in deionized water as described [15].

### 2.2. Animal Model

All experiments involving animals were carried out in accordance with the United States National Institutes of Health Guide for the Care and Use of Laboratory Animals and were approved by the University of Louisville Institutional Animal Care and Use Committee.

Eight-week-old male C57BL/6J mice were obtained from the Jackson Laboratory (Bar Harbor, Maine) and housed in the University of Louisville Research Resources Center at 22°C with a 12-hour light/dark cycle. Forty mice were randomly assigned to 8 groups (*n* = 5) and fed by either normal diet (ND, 10 Kcal% fat; D12450B, Research Diets Inc., 3.85 Kcal/g) or by HFD (60 Kcal% fat; D12492, Research Diets Inc., 5.24 Kcal/g). Simultaneously, these mice were daily gavage-administered with vehicle (0.5% ethanol), BL153 (5 mg/kg), low dose MH (L-MH, 0.5 mg/kg, equal to 5 mg/kg BL153), or high dose MH (H-MH, 1.0 mg/kg), respectively, for 24 weeks. Daily food intake and weekly body-weight were monitored. Gavage volume was 1% of mouse body-weight and adjusted based on the body-weight change.

### 2.3. Intraperitoneal Glucose Tolerance Test (IPGTT)

IPGTT was performed on the 23rd week of ND or HFD feeding [27]. After a 6-hour fast (8:00 am–2:00 pm), mice were injected intraperitoneally with D-(+)-glucose (Sigma-Aldrich, St. Louis, MO, USA) by 2 g/kg body-weight. Blood glucose levels at 0, 15, 30, 60, and 120 min after glucose injection were measured using a FreeStyle Lite glucometer (Abbott Diabetes Care, Alameda, CA). Area under the curve (AUC) for the glucose tolerance curve was calculated by the trapezoid rule.

### 2.4. Body Fat Analysis

Mouse body fat mass was assessed using DEXA scan (PIXImus2, Lunar, Madison, WI) as previously reported [28]. The mice were anesthetized by inhalation of 2-3% isoflurane-oxygen gas via nose cone and then placed on the scanner bed in the prone position with the limbs and tail stretched away from the body. The body fat mass was scanned with dual energy X-ray absorptiometry at the 24th week of the experiment.

### 2.5. Sample Collection and Chemicals Quantification

After performance of glucose tolerance test and body fat mass scan, mice were fasted for 6 h and then sacrificed for further analysis at the end of 24th week study. Blood samples were collected for blood glucose, plasma insulin (Crystal Chem Inc., Downers Grove, IL), triglyceride (TG), and cholesterol (Cayman Chemical, CA) levels and alanine transaminase (ALT) activity (Thermo scientific, VA) measured as described by the manufacturers. Homeostasis model assessment-estimated insulin resistance (HOMA-IR) index is calculated by using both fasting glucose and insulin as follows: HOMA-IR = glucose × insulin/405, where glucose is given in mg/dl and insulin is given in *μ*U/mL [29]. Two sides of epididymal white adipose tissue (WAT) and whole liver were isolated, weighted, and saved for further analysis. Mouse tibia length was measured for obese index calculation.

### 2.6. Histological Analysis

Fixed WAT and liver tissues were routinely embedded in paraffin, sectioned, and then performed common H & E staining for conventional histopathological examination with optical microscope (Nikon, Melville, NY); acidified toluidine blue staining for mast cells was done for WAT tissue slides with potassium permanganate solution for 2 min., potassium metabisulphite solution for 1 min., and then in acidified toluidine blue solution for 5 min. at room temperature [30]. Mast cells were captured by the optical microscope.

### 2.7. Statistical Analysis

Data were collected from 5 animals per group and presented as means ± SEM as indicated. Comparisons were performed by one-way ANOVA for the different groups, followed by post hoc pairwise repetitive comparisons with Tukey's test using Origin 8.6 Lab data analysis and graphing software (Origin Lab Corporation, Northampton, MA). Statistical significance was considered as *P* < 0.05.

## 3. Results

### 3.1. Effects of MH on Body-Weight and Food Intake

**Figure 2:**
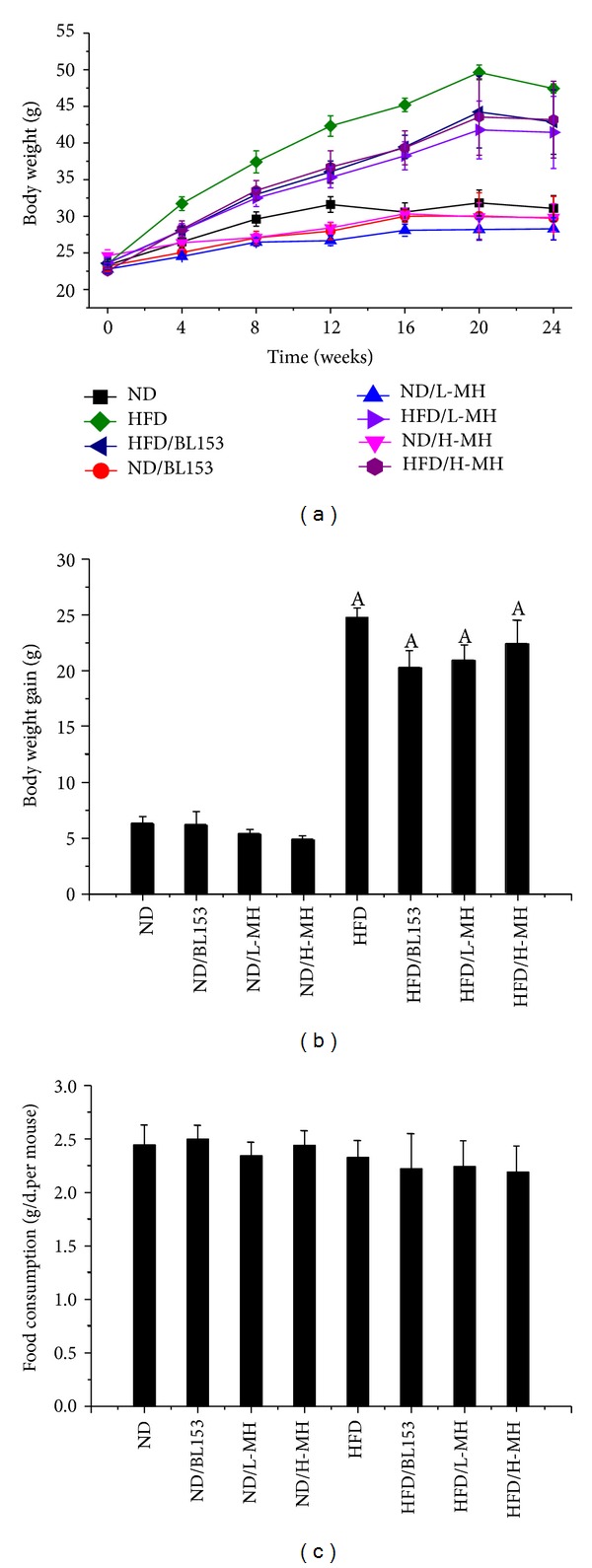
Effects of MH on body-weight and food intake in mice fed by normal fat diet (ND) or by high-fat- diet (HFD). Male C57/BL/6J mice at 8 weeks of age were fed either by ND (10% kcal as fat) or by HFD (60% kcal as fat) with vehicle (0.5% ethanol), BL-153 (5 mg/kg body weight), or MH (0.5 or 1 mg/kg body-weight) for 24 weeks. (a) Time-course body-weight change; (b) body-weight gain at the end of experiment; (c) average food intake for 12 weeks. Data were presented as means ± SEM (*n* = 5). A:  *P* < 0.05 versus ND.

In the study, the effect of MH on body-weight as a character of obesity was measured. Compared to ND, HFD feeding gradually increased body-weight (Figures 2(a) and 2(b)) although the food intake did not show obvious difference (Figure 2(c)). MH treatment exhibited a tendency to inhibit the body-weight gain without statistically significant difference compared to HFD group (Figure 2(b)).

### 3.2. Effects of MH on Body Fat Mass, Lipid Accumulation, and Anti-Inflammation in Adipose Tissue

**Figure 3:**
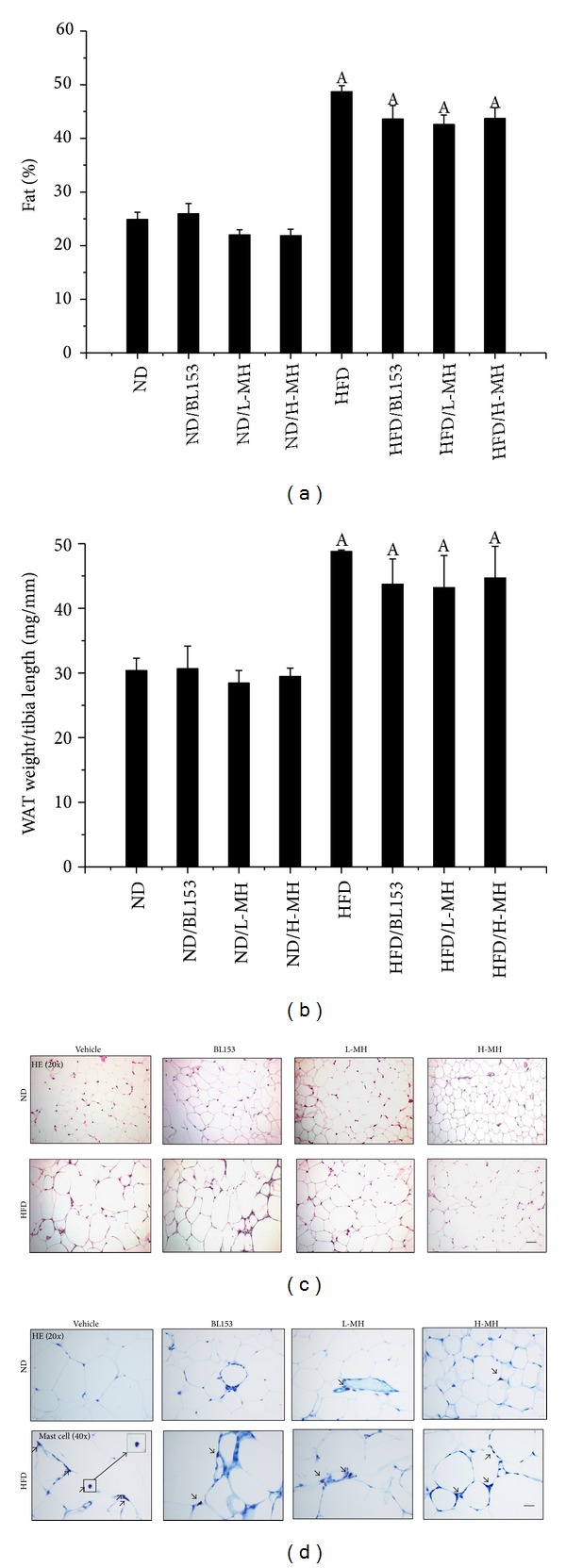
Effects of MH on body fat composition and epididymal white adipose tissue (WAT). (a) Body fat mass was measured; (b) epididymal WAT weight was normalized by tibia length; (c) adipose histology was examined by using hematoxylin and eosin (H & E) staining (magnifications of ×20); (d) acidified toluidine blue staining for mast cells (arrow pointed) (magnifications of ×40). Data were presented as means ± SEM (*n* = 5). A:  *P* < 0.05 versus ND; bar = 50 *μ*m.

Similarly HFD significantly increased whole-body fat content up from 27% to 48% of ND feeding. MH treatment slightly reduced the fat composition (~43%) after 24 weeks administration (Figure 3(a)). The result of epididymal white adipose tissue (WAT) weight normalized by tibia length was similar to whole-body fat content (Figure 3(b)). H & E staining showed that the adipose cells were enlarged due to more fat deposit in adipocytes in HFD-fed mice compared to those in ND-fed mice. H-MH treatment obviously lowered the adipocyte size (Figure 3(c)). Obesity is a chronic inflammatory state characterized by infiltration of adipose tissue by immune cell populations. As one of immune cells, mast cells were regarded as cellular actors involved in the pathophysiology of obesity by white adipose tissue and systemic inflammation. In the experiment, the mast cells in WAT stained by acidified toluidine blue were observed. Both MH and BL153 treatments visibly decreased mast cell infiltration to WAT (Figure 3(c)).

### 3.3. Effects of MH on Plasma Triglyceride and Cholesterol

**Figure 4:**
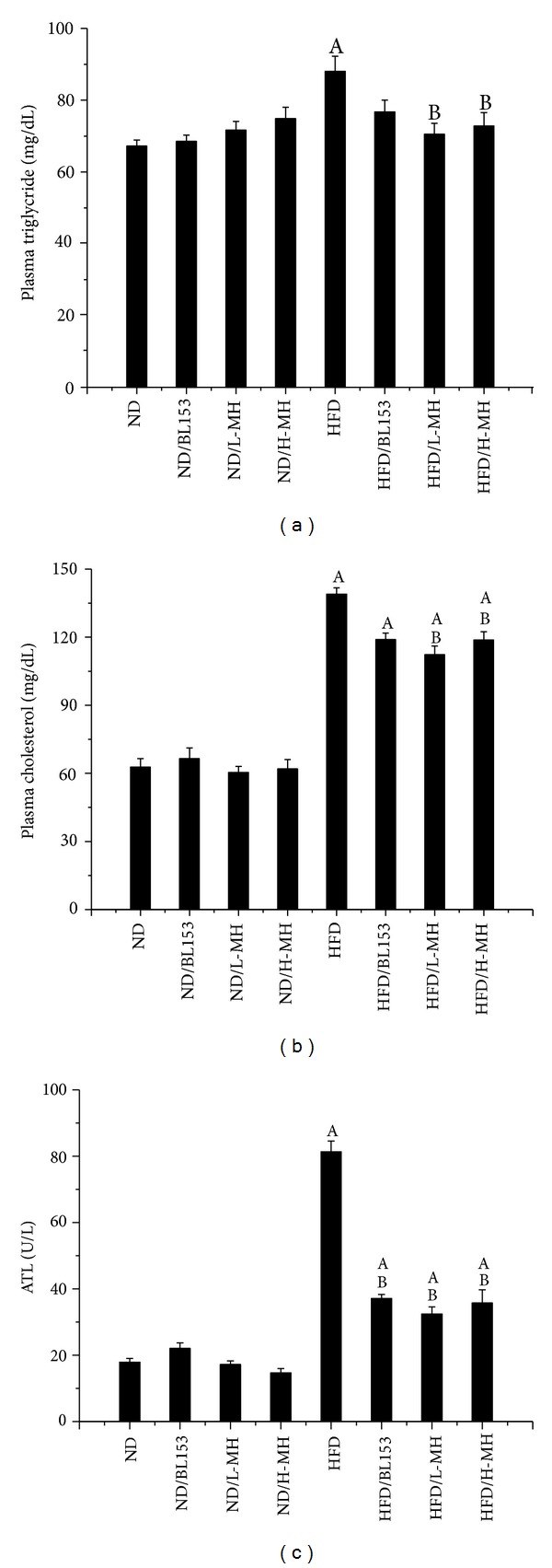
Effects of MH on plasma triglyceride (a), cholesterol (b), and alanine transaminase ALT (c). Data were presented as means ± SEM (*n* = 5). A: *P* < 0.05 versus ND; B: *P* < 0.05 versus HFD.

Hyperlipidemia is one of the characters in HFD-induced obesity in mice. It has long been recognized that plasma TG and cholesterol concentrations are commonly elevated in obese individuals. We measured the TG and cholesterol levels in plasma. Both L-MH and H-MH treatments significantly reduced the HFD-induced increases of TG (Figure 4(a)) and cholesterol (Figure 4(b)) levels in plasma. BL153 treatment showed a reduced tendency but no significant difference compared to HFD-fed mice (Figure 4).

### 3.4. Effects of MH on Hepatic Steatosis

**Figure 5:**
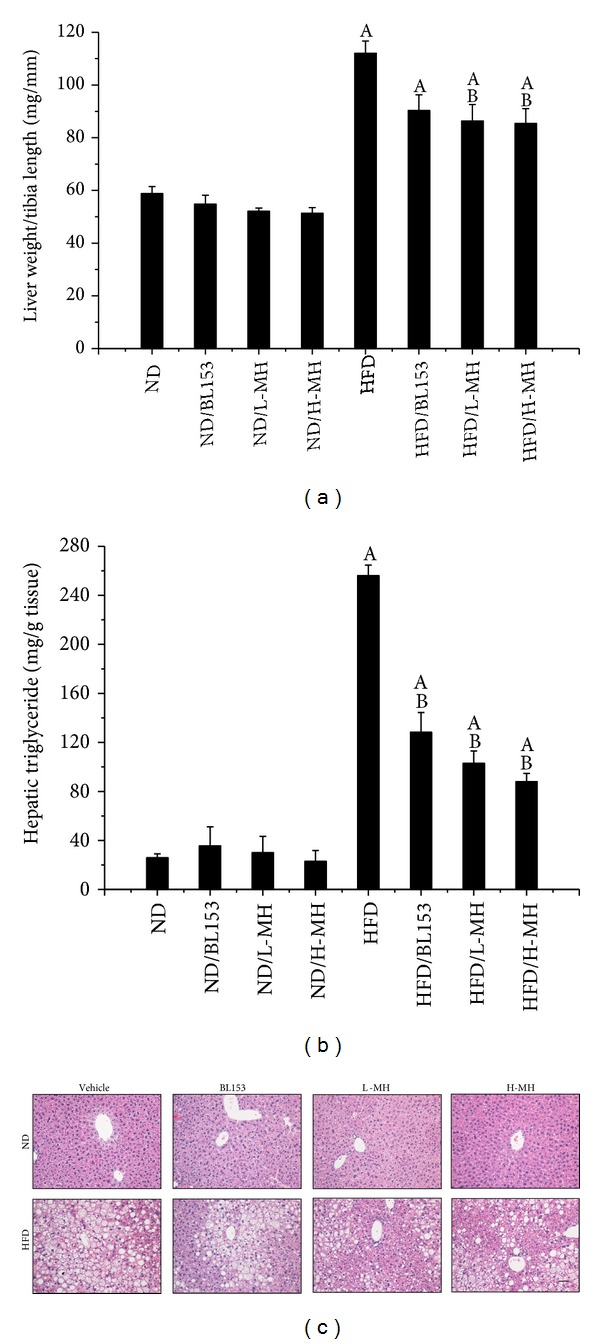
Effects of MH on the liver weight, lipid accumulation, and steatosis. (a) Liver weight was normalized by tibia length; (b) hepatic TG level was measured by a TG assay kit; (c) H & E staining for hepatic structure (magnifications of ×20). Data were presented as means ± SEM (*n* = 5). A: *P* < 0.05 versus ND; B: *P* < 0.05 versus HFD; bar = 50 *μ*m.

Liver is also a main target for HFD-induced obesity. In the experiment, HFD feeding significantly increased liver weight normalized by tibia length as well as hepatic TG level. MH treatment significantly lowered liver weight (Figure 5(a)) and showed a dose-dependent decrease of hepatic TG (Figure 5(b)). BL153 obviously reduced hepatic TG and slightly diminished liver weight (Figures 5(a)and 5(b)). Histological analysis showed that HFD-induced hepatic steatosis was improved by both MH and BL153 treatments for 24 weeks (Figure 5(c)). As an indicator of the severity of liver injury, markedly augmented ALT in plasma was inhibited by both MH and BL153 treatments in HFD-fed mice (Figure 4(c)).

### 3.5. Effects of MH on Systemic Insulin Resistance

**Figure 6:**
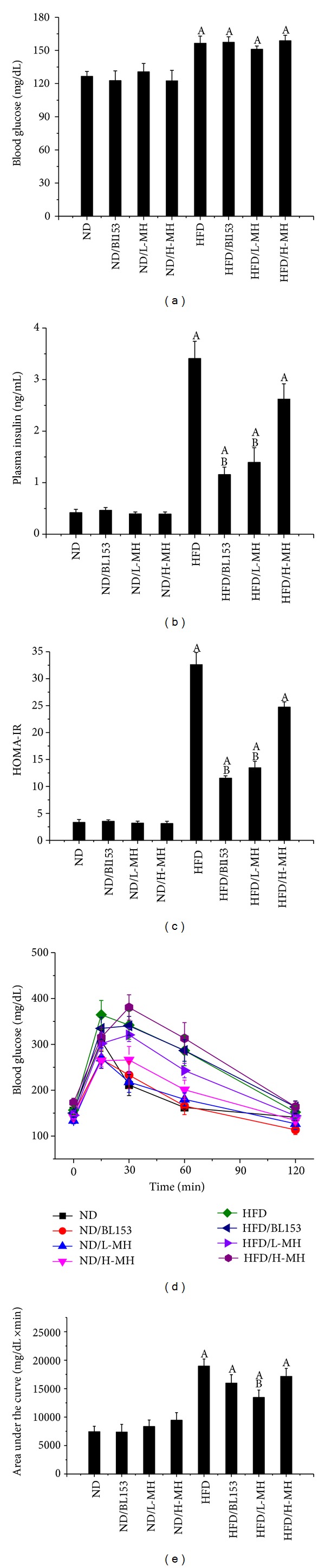
Effects of MH on the glucose tolerance, blood glucose, and plasma insulin level in mice fed with ND or with HFD for 24 weeks. (a) Blood glucose was measured using a glucometer. (b) Plasma insulin level was measured by kit. (c) HOMA-IR was calculated by using both fasting glucose and insulin as follows: HOMA-IR = glucose × insulin/405, where glucose is given in mg/dl and insulin is given in *μ*U/mL. (d) Blood glucose concentration during glucose tolerance test (2 g/kg) following fasting 6 h in mice. (e) The area under the curve (AUC) for glucose tolerance was calculated using the trapezoidal rule. Data were presented as means ± SEM (*n* = 5). A: *P* < 0.05 versus ND; B: *P* < 0.05 versus HFD.

We investigated whether MH affects HFD-induced insulin resistance, showing that HFD feeding increased fasting blood glucose (Figure 6(a)) and plasma insulin levels (Figure 6(b)), consistent with previously published data [31]. Although MH or BL153 treatment did not decrease HFD feeding-induced fasting glucose raise (Figure 6(a)), L-MH and BL153 treatment resulted in a significant reduction of fasting plasma insulin (Figure 6(b)) and HOMA-IR (Figure 6(c)). In IPGTT, the level of area under the curve was obviously diminished in L-MH treatment mice compared to HFD feeding. H-MH and BL153 slightly improved this effect without statistically significant difference (Figures 6(d) and 6(e)).

## 4. Discussion 

Obesity is a whole-body system derangement. Diet-induced obese model results in multiple organ changes, such as adipocyte hypertrophy, hepatic steatosis, hyperinsulinemia, and insulin resistance [4]. In the present study, we observed that long-term HFD feeding increased body-weight, body fat mass, adipocyte size, and abnormal infiltration of mast cells in adipose tissue. Lipid accumulation in the liver leads to hepatic TG increase, steatosis, and liver injury. Damaged hepatocytes released amount of ALT to the blood stream. HFD-induced obesity exhibited high glucose, insulin, TG and cholesterol in the blood, and insulin resistance. Treatment of MH or* Magnolia* extract BL153 amended to a certain extent these phenotypes even if not completely returned back to normal as those of ND feeding. Despite slight decrease in body-weight (Figure 2) and fat composition (Figure 3), MH significantly lowered plasma TG and cholesterol levels (Figure 4), markedly ameliorated insulin resistance (Figure 6) and hepatic steatosis (Figure 5), along with decrease of liver weight, hepatic TG level (Figure 5(b)), and ALT activity (Figure 4(c)), and obviously deceased adipocyte size and amount of mast cells in adipose tissue (Figures 3(c) and 3(d)).

Obesity is caused by an excessive intake of calories for a long period of time. Several studies have demonstrated that long-term HFD feeding leads to mouse obesity characterized by body-weight and fat mass increase [4–6, 32]. TG, as the principal component of dietary fat, increases the palatability and energy density of food. Increased dietary fat intake is associated with increases in total energy intake and body- weight, which may explain the results in the study that the body-weight increased in HFD-fed mice without alteration in the amount of food consumption compared to ND-fed mice. White adipose tissue (WAT) is the primary storage for excessive amounts of TG. Adipocyte hypertrophy and hyperplasia were observed in HFD-induced obesity animal models [32]. C57BL/6J mice-fed HFD with supplement of HK or MG for 16 weeks lowered the weight of WAT as well as adipocyte size. This change was related to a significant increase in energy consumption and activities of fatty acid *β*-oxidation and carnitine palmitoyltransferase and decrease in activity of fatty acid synthesis (FAS) and in the mRNA expression of key genes involved in FAS as well as adipocyte differentiation in epididymal WAT [32]. In our study, we only observed a slight decrease of WAT weight and visible smaller adipocyte size in MH or BL153-supplemented mice (Figures 2 and 3). MH and BL153 may promote the activities of enzymes related to lipolysis.

Obesity is associated with lipid accumulation not only in adipose tissue but also in nonadipose tissue [33]. Liver plays an important role in maintaining glycogen homeostasis, plasma protein synthesis, and drug detoxification. It is also a central place for trafficking of lipids, such as synthesizing cholesterol and TG, producing and taking up lipoproteins. Insulin stimulates fatty acid and TG synthesis in liver, and then these products were released to blood stream. Therefore, hepatocytes are capable of storing lipids as small droplets of TG. Normally, hepatic lipid content is below 5% by liver weight [33]. Long-term HFD will lead to hepatic steatosis due to excess lipids accumulation in liver, which is associated with the group of metabolic abnormalities characterized by an increase in intrahepatic TG content and insulin resistance [33]. As a biomarker of liver injury, ALT elevation is related to alterations in insulin sensitivity, glucose tolerance, and inflammation [34]. Hyperlipidemia is also one of the main characters in HFD-induced obesity. In the study we observed that HFD feeding significantly increased liver weight, hepatic TG levels, and plasma TG, cholesterol, and ALT activities compared to ND feeding (Figures 4 and 5). Correspondingly, more hepatic lipid droplets were assessed in HFD-fed mice than in ND-fed mice (Figure 5(c)). Treatment with MH or BL153 significantly attenuated plasma ALT activities and hepatic TG. Moreover, MH treatment also markedly reduced fasting plasma TG and cholesterol, liver weight, and hepatic TG after HFD feeding (Figures 4 and 5). These results suggested that MH has relatively stronger ability to antilipid accumulation than BL153 in the model.

Obesity is a chronic inflammatory state. It is proved that the inflammation in obese adipose tissue could enhance metabolic disorders, such as insulin resistance and hepatic steatosis [35].* Magnolia* extract and its active component MG were found to prevent skin photoaging via inhibition of NF-*κ*B in mice [36]. Intraperitoneal treatment of MH significantly inhibited acetic acid-induced acute inflammation and carrageenan-induced paw swelling and tissue plasminogen activator induced increase in ear thickness and ear weight [37]. In the study, histological staining showed that mast cells in adipose tissue were obviously reduced in HFD-induced obesity with MH or BL153 treatment (Figure 3(d)). Poglio et al. found that WAT contained a significant mast cell progenitor population. The entire mast cell lineage process took place in WAT [38]. Therefore, HFD-induced excess FA storage in adipose tissue might trigger the progenesis of mast cell. MH and BL153 could inhibit this process in adipose tissue.

Generally, the elevation of fasting glucose is accompanied by increases in fasting insulin level [4]. We found that fasting blood glucose, fasting plasma insulin, and insulin resistance which were evaluated by the cumulative changes in blood glucose responses quantified by the incremental area under the curve in IPGTT test were significantly increased in HFD-fed mice. Treatment of MH significantly ameliorated HFD-induced insulin resistance presented by reducing fasting plasma insulin and the area under the curve in IPGTT, and BL153 slightly improved insulin resistance and significantly reduced fasting plasma insulin (Figure 6), which was consistent with the results in our previous study [15].

In summary, this study demonstrated that MH treatment ameliorated HFD-induced lipid accumulation and inflammation in adipose tissue, hepatic steatosis, and insulin resistance in obese mice. Compared to the selected reference BL153, MH was relatively more efficient. Therefore, MH may have a potential benefit to protect HFD-induced obesity.
